# PAC proton-activated chloride channel contributes to acid-induced cell death in primary rat cortical neurons

**DOI:** 10.1080/19336950.2020.1730019

**Published:** 2020-02-25

**Authors:** James Osei-Owusu, Junhua Yang, Maria Del Carmen Vitery, Mengnan Tian, Zhaozhu Qiu

**Affiliations:** a Department of Physiology, Johns Hopkins University School of Medicine, Baltimore, MD, USA; b Solomon H. Snyder Department of Neuroscience, Johns Hopkins University School of Medicine, Baltimore, MD, USA

**Keywords:** Proton-activated chloride channel, TMEM206, PACC1, acid-induced neuronal death, ischemic stroke

## Abstract

Severe local acidosis causes tissue damage and pain, and is associated with many diseases, including cerebral and cardiac ischemia, cancer, infection, and inflammation. However, the molecular mechanisms of the cellular response to extracellular acidic environment are not fully understood. We recently identified a novel and evolutionarily conserved membrane protein, PAC (also known as PACC1 or TMEM206), encoding the proton-activated chloride (Cl^−^) channel, whose activity is widely observed in human cell lines. We demonstrated that genetic deletion of *Pac* abolished the proton-activated Cl^−^ currents in mouse neurons and also attenuated the acid-induced neuronal cell death and brain damage after ischemic stroke. Here, we show that the proton-activated Cl^−^ currents are also conserved in primary rat cortical neurons, with characteristics similar to those observed in human and mouse cells. *Pac* gene knockdown nearly abolished the proton-activated Cl^−^ currents in rat neurons and reduced the neuronal cell death triggered by acid treatment. These data further support the notion that activation of the PAC channel and subsequent Cl^−^ entry into neurons during acidosis play a pathogenic role in acidotoxicity and brain injury.

## Introduction

Chloride (Cl^−^) is the most abundant free anion in animal cells. Cl^−^ channels play important roles in diverse physiological functions, including cell and organelle volume regulation, fluid secretion, vesicular acidification, and regulation of electrical excitability [1,2]. However, despite recent progress, Cl^−^ channels are considerably under-studied compared to cation (Na^+^, K^+^, and Ca^2+^) channels. Many electrophysiologically well characterized Cl^−^ channels still lack molecular identity [2]. This gap makes it impossible to elucidate their precise biological function and how their dysfunction contributes to various diseases. The proton-activated Cl^−^ channel, opened by exposure to extracellular acidic environment, is such an example [3]. It is also previously referred to as PAORAC (proton-sensitive outwardly rectifying anion channel) [4] or ASOR (acid-sensitive outwardly rectifying anion channel) [5]. Through a cell-based fluorescence assay for Cl^−^ channels and an unbiased RNA interference (RNAi) screen, we recently identified a novel two-transmembrane protein PAC (also known as PACC1 or TMEM206) as being essential for the proton-activated Cl^−^ (PAC) currents in human cell lines [6]. PAC shares no significant sequence similarity to any other known ion channels. Overexpression of human PAC cDNA in *PAC* knockout cells generated large proton-activated Cl^−^ currents with the same characteristics as the endogenous ones, including a strong outwardly rectifying current-voltage relationship, time-dependent facilitation at positive membrane potentials, and a low field strength anion permeability sequence (I^−^> Br^−^ > Cl^−^). Further studies on the ion channel properties of human PAC with point mutations and fish PAC suggested that PAC directly forms the proton-activated Cl^−^ channel pore [6]. Using a very similar approach, another independent study also identified PAC (TMEM206) as the proton-activated Cl^−^ channel [7].

Tissue acidosis is a wide-spread pathological feature associated with many diseases, including cancer, ischemia, infection, and inflammation [8–12]. It contributes to tumor progression, tissue damage, and pain. Although the threshold to elicit the proton-activated Cl^−^ currents is relatively low, ~pH 5.5 at room temperature, the channels become more sensitive and are activated under less acidic conditions of ~pH 6.0 at body temperature (37°C) [6,13,14]. Local brain tissue pH can fall below 6.0 during ischemic stroke, in which acidosis is one of the important mechanisms promoting neuronal death and brain injury [15–18]. Due to the low intracellular Cl^−^ concentration in mature neurons, opening of the PAC channel is proposed to mediate Cl^−^ influx, leading to subsequent cell swelling and cell death [5,14]. Consistent with this hypothesis, knockout of mouse *Pac* abolished the proton-activated Cl^−^ currents in primary cortical neurons [6]. Furthermore, *Pac* knockout mice exhibited smaller brain infarct volume with improved neurological scores 24 hours after the permanent middle cerebral artery occlusion (pMCAO) [6], a widely performed ischemic stroke animal model [19]. However, it’s unknown whether the involvement of PAC in acid-induced neuronal injury is a general phenomenon, or if it is just limited to mouse. In this study, we asked whether the proton-activated Cl^−^ currents are conserved in rat neurons, and if the rat PAC channel also contributes to neuronal cell death triggered by acidosis.

## Results and discussion

**Figure 1.**
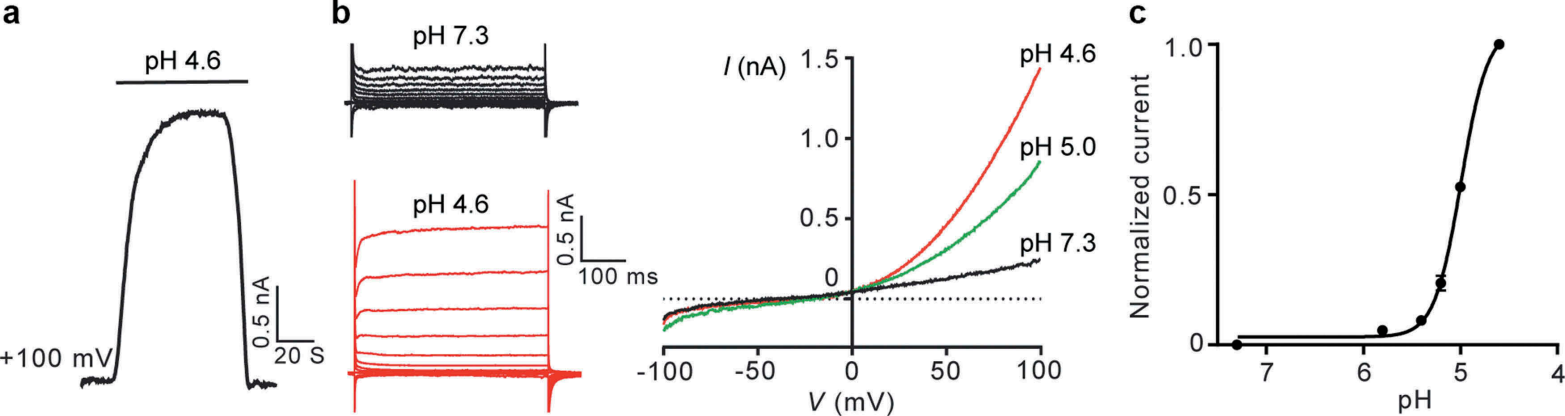
Characterization of the proton-activated Cl^−^ currents in primary rat cortical neurons. (a), Typical time course of extracellular pH 4.6-stimulated whole-cell currents for primary rat cortical neurons. (b), Representative whole-cell currents at different pH are monitored by voltage step (left) and ramp (right) protocols. (c), Normalized currents (background-subtracted current at pH 4.6 and +100 mV as 1, *n* = 6–8 cells) to pH relationship at room temperature fitted in a Hill equation.

To examine whether the proton-activated Cl^−^ currents are conserved in rat cells, we performed whole-cell patch clamp recording in primary rat cortical neurons. Perfusion of acidic bath solutions elicited large and stable conductances at +100 mV membrane potential (Figure 1(a)). The low pH-induced currents exhibited slight time-dependent facilitation at positive membrane potentials (Figure 1(b)). The current-voltage relationships displayed a steep outward rectification (Figure 1(b)). We further fitted the normalized current-to-pH relationship with a Hill equation (Figure 1(c)). This yielded a pH_50_ of 5.0 and a Hill coefficient of 4, suggesting current activation by cooperative binding of protons. These electrophysiological properties are characteristic of the proton-activated Cl^−^ currents observed in human cell lines [3,6].

**Figure 2.**
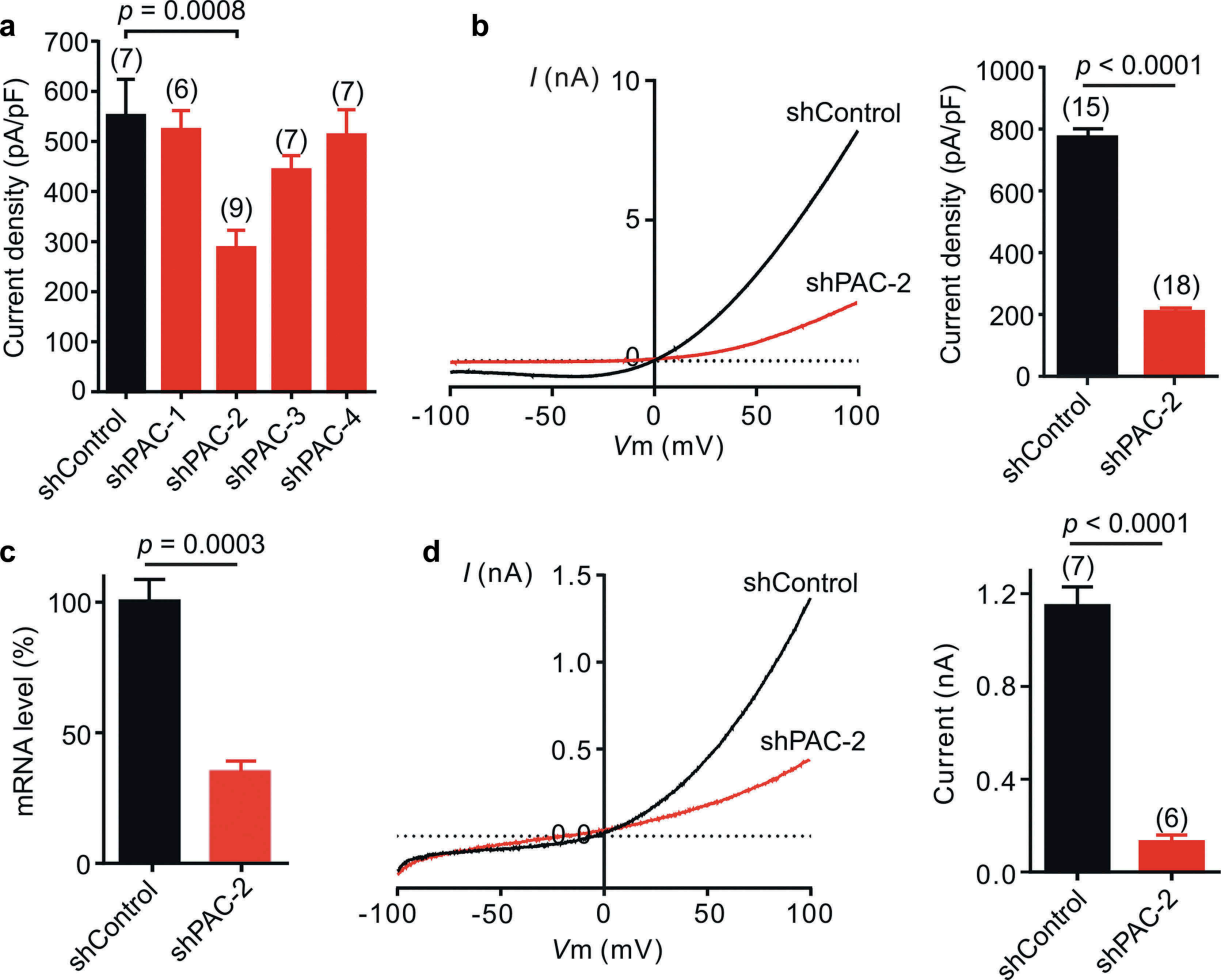
Knockdown of Pac markedly reduced the proton-activated Cl^−^ currents in rat cells. (a), Screening for shRNA targeting rat *Pac* in rat INS-1E cells by measuring extracellular pH 4.6-induced current densities (mean ± SEM) at +100 mV 3 days after shRNA transfection (one-way analysis of variance (ANOVA) with Bonferroni post hoc test). (b), Example (left) and quantification (right) of pH 4.6-induced currents for rat INS-1E cells 4 days after shRNA transfection. Bars represent mean ± SEM (two-tailed Student’s *t* test). (c), shPAC-2-mediated *Pac* mRNA knockdown (mean ± SEM, *n* = 4 biological replicates) in primary rat cortical neurons assayed by qPCR. Expression levels were normalized to cells transduced with control shRNA as 100%. (d), Representative whole-cell currents at pH 4.6 monitored by voltage ramp protocol for shPAC-2-treated primary neurons. Background-subtracted current densities (mean ± SEM, two-tailed Student’s *t* test) at pH 4.6 and +100 mV for shPAC-2-treated primary rat neurons.

Rat PAC shares 91% amino acid identity with human PAC [6]. To test whether PAC is responsible for the proton-activated Cl^−^ currents in rat neurons (Figure 1), we designed 4 short-hairpin RNAs (shRNAs) targeting rat *Pac* gene. Using patch clamp recording, we screened the inhibitory efficiency of lentivirus-transduced shRNAs in rat insulinoma INS-1E cells, which expressed large proton-activated Cl^−^ currents (Figure 2(a)). Compared to control shRNA, shPAC-2 significantly reduced the current density 3 days after lentiviral transduction (Figure 2(a)), with an even more dramatic suppressive effect after 4 days (Figure 2(b)). We next transduced primary rat neurons with lentiviruses expressing shPAC-2 at 6 days in vitro (DIV 6). Consistently, shPAC-2 knocked down the *Pac* mRNA expression level (Figure 2(c)) and markedly reduced the proton-activated Cl^−^ currents recorded at DIV 9 (Figure 2(d)). These experiments with INS-1E cells and primary cortical neurons indicate that the proton-activated Cl^−^ channel is expressed in different rat tissues and is dependent on PAC expression.

**Figure 3.**
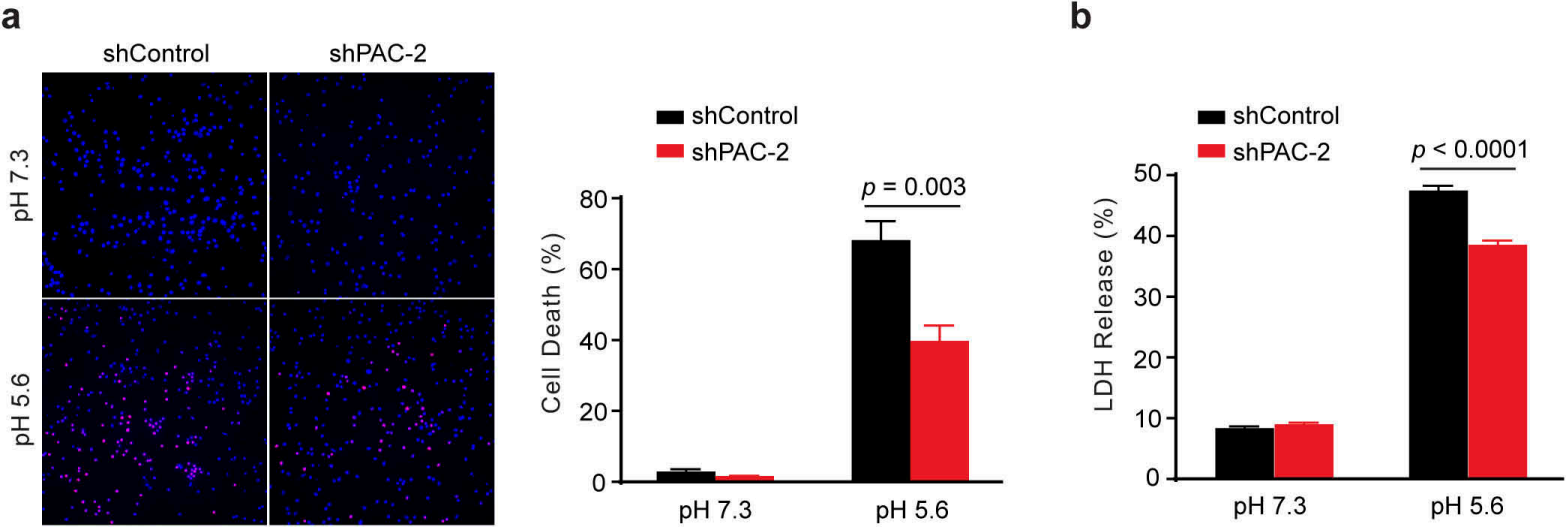
*Pac* knockdown attenuates acid-induced cell death of primary rat cortical neurons. (a), Representative images (left) and quantification (right; mean ± SEM, *n* = 5 coverslips for each group, two-tailed Student’s *t* test) of acid-induced cell death in control and shPAC-2-treated primary neurons (1-hour pH 7.3 or 5.6 treatment and 24-hour recovery in culture medium), stained with Hoechst 33342 (blue) for total nuclei and Propidium iodide (red) for dead neurons at 20x magnification. (b), Quantification (mean ± SEM, *n* = 3 wells for pH 7.3 and *n* = 6 wells for pH 5.6, two-tailed Student’s *t* test) of acid-induced cell death by the percentage release of LDH from rat cortical neurons (1-hour pH 7.3 or 5.6 treatment and 24-hour recovery in culture medium), normalized to total LDH.

To further test whether PAC is involved in acid-induced neuronal cell death, we treated rat primary cortical neurons with neutral (pH 7.3) or acidic (pH 5.6) solution for 1 hour. After 24-hour recovery in culture medium, we stained the coverslips with Hoechst 33342 (blue) for nuclei of all neurons and with propidium iodide (red) for nuclei of dead neurons [9]. Minimal cell death in the *Pac* shPAC-2-transduced neurons was observed at neutral pH (Figure 3(a)), suggesting that PAC is not required for cell survival under normal conditions. As expected, acid treatment caused massive cell death in control shRNA-transduced neurons (Figure 3(a)). However, the percentage of cell death was significantly reduced in *Pac* knockdown neurons (Figure 3(a)). The role of PAC in cell viability during the acid treatment was further investigated using the release of a cytosolic enzyme lactate dehydrogenase (LDH) as a measure of cell death [9,18]. Again, acid-induced neuronal cell death, as indicated by LDH release, were also partially reduced by shPAC-2-mediated *Pac* knockdown (Figure 3(b)). These data indicate that the PAC channel contributes to acid-induced cell death in primary rat cortical neurons.

Here we show that the proton-activated Cl^−^ currents are conserved in rat cells with very similar properties to those observed in human cells. In addition to the previous reports in the mouse with genetic deletion and channel inhibitors [6,14], a pathogenic role of the PAC channel in acidotoxicity is further supported by the current study using primary rat cortical neurons. The activation threshold of PAC is relatively low (~pH 5.5 at room temperature), however, it is possible that the PAC channel may be modulated by yet unknown intra- or extracellular signals (as shown for temperature) and becomes more readily activated under less acidic environments in diseases, such as ischemic stroke. The molecular identification of the PAC channel will facilitate the identification of physiological/pathological and small molecule PAC modulators. The Na^+^-permeable acid-sensing ion channels (ASICs) were also shown to be involved in necrotic neuronal cell death under acidic conditions both *in vitro* and *in vivo* [8,17]. Therefore, it is a possibility that activation of ASICs gives rise to depolarization, which facilitates Cl^−^ entry via the simultaneously activated PAC channel. The influx of NaCl through both channels drives water inflow, resulting in the neuronal swelling and cell death [18]. Further study will test this hypothesis and examine whether there are synergistic benefits of inhibiting both acid-sensitive ion channels in ischemic stroke and other diseases associated with acidosis.

## Methods

### Electrophysiology

Whole-cell patch clamp recordings were done as previously described [6]. Primary rat cortical neurons for whole-cell recordings were cultured onto poly-D-lysine-coated coverslips. The standard bath solution contained (in mM) 145 NaCl, 2 KCl, 2 MgCl_2_, 1.5 CaCl_2_, 10 HEPES, 10 glucose (300 mOsm/kg; pH 7.3 with NaOH). Low pH solutions were made of the same ionic composition with 5 mM Na_3_-citrate as buffer instead of HEPES, and the pH was adjusted using citric acid. All patch clamp recordings were performed using borosilicate glass pipettes, pulled with a Model P-1000 multi-step puller (Sutter Instruments). Recording pipettes had a resistance of 2–5 MΩ when filled with an internal solution containing (in mM): 135 CsCl, 1 MgCl_2_, 2 CaCl_2_, 10 HEPES, 5 EGTA, 4 MgATP (280–290 mOsm/kg; pH 7.2 with CsOH). Cells were continuously perfused with extracellular solutions using a gravity perfusion system. Recording data were acquired at room temperature using MultiClamp 700B amplifier and 1550B digitizer (Molecular Devices). Data were collected at 10 kHz sampling rate and filtered at 2 kHz. Voltage-dependent activation recordings were done by holding cells at −60 mV and applying voltage step pulses (500 ms duration, 5 s interval) from −100 to +100 mV in 20 mV increments. The ramp protocol for current-voltage relationship was from −100 to +100 mV at a ramp speed of 1 mV/ms, with 4 s time interval between ramps. Clampfit 10.6 and GraphPad Prism 6 were used for all data analyses. Background currents at pH 7.3 were subtracted from currents at acidic pH to plot the pH curve and current densities.

### Cell culture

All cells were cultured and maintained at 37°C in a 5% CO_2_ atmosphere. Rat insulinoma INS-1E cells were obtained from ATCC (a kind gift from Dr. Dax Fu, Johns Hopkins University) and propagated in complemented RMPI 1640 medium supplied with 100 units/ml penicillin (P), 100 µg/ml streptomycin (S), 10% (v/v) fetal bovine serum (FBS), 2 mM glutamine, 10 mM HEPES, 1 mM sodium pyruvate and 50 µM 2-mercaptoethanol. HEK293 cells were maintained in Dulbecco’s modified Eagle’s medium (DMEM) supplemented with 10% FBS and 1% P/S.

Primary cortical neurons were prepared from E18 Sprague Dawley rat embryos, as described previously [20]. In brief, dissected cerebral cortices were enzymatically digested and gently triturated to dissociate cells. Cells were then seeded on poly-D-lysine coated plates or coverslips. The culture medium was half changed every 3 days with neurobasal medium (Gibco) containing B27 supplement and glutamine. Astrocyte proliferation was inhibited by adding 5 µM FDU (5-Fluoro-2ʹ-deoxyuridine) at 3 days in vitro (DIV3).

### Lentiviral shRNA transduction

Four shRNA sequences targeting rat *Pac* gene were designed (Invitrogen BLOCK-It RNAi Designer) and inserted into Pll3.7 vector (Addgene): #1 GCTCCAGGGATTGCCTTATAC; #2 GCACCACTCAGAGGATCAATT; #3 GCGTTCCTGGCATTATTTAAA; #4 GCCAGGCGACAAACCACATAA. ShRNA targeting luciferase GL-3 was used as control. The knockdown efficiency was evaluated by transiently transfecting rat INS-1E cells and recording the proton-activated Cl^−^ currents. Transfection was done using Lipofectamine 2000 (Life Technologies) according to the manufacturer’s instruction. shPAC-2 was selected as a potent shRNA (Figure 2). Lentiviruses were produced by co-transfecting HEK293 cells with shRNA constructs, pMD2.G, pRSV-REV and pMDL.RRE following Addgene’s instruction. Primary rat cortical neurons were transduced at DIV6 and infected cells (GFP+) were recorded 3–4 days after viral transduction. The knockdown efficiency was assessed by qPCR.

### RNA isolation and quantitative real-time PCR

Total RNA from transduced cells was first isolated using TRIzol reagent (Life Technologies). RNA was reverse transcribed to cDNA using qScript XLT cDNA SuperMix (Quanta Biosciences). PrimeTime qPCR assays for rat *Pac* (assay id: Rn.PT.58.10685490) and rat *Actb* (assay id: Rn.PT.39a.22214838.g) were obtained from Integrated DNA Technologies. All reactions were performed in triplicates using the PerfeCTa FastMix II, low ROX (Quanta Biosciences). The reaction was run according to the manufacturer’s protocol in the QuantStudio 6 Flex Real-time PCR system. *Actb* was used as the reference gene and all qPCR results were analyzed and normalized using the 2^−∆∆CT^ method.

### Acid-induced cell death

Primary rat cortical neurons, 4 days after viral transduction, were treated with pH 7.3 or 5.6 solution for 1 hour at 37°C. After 24-hour recovery in culture medium, the neurons were stained with 10 µg/ml propidium iodide (PI) and 1 mg/ml Hoechst 33342 (Life Technologies) for 10 min at room temperature. Cells were then fixed with 4% paraformaldehyde. Coverslips were mounted and imaged with Zeiss Axiophot fluorescent microscope. Cells were counted (>2,500 cells per coverslip) using NIH ImageJ software. Percentage of dead cells was calculated from the ratio of the PI positive nuclei (for dead neuron nuclei) to Hoechst 33342 positive nuclei (for total neuron nuclei).

For the lactate dehydrogenase (LDH) assay, cell death was measured by quantifying LDH release. After 1-hour acid treatment and 24-hour recovery, neurons with damaged membranes release the LDH enzyme into the culture medium. The LDH assay was done using the CytoTox 96 Non-Radioactive Cytotoxicity Assay (Promega), according to the manufacturer’s instruction. Culture supernatants were incubated with the LDH cytotox 96 reagent for 30 min and absorbance at 490 nm was read in three technical replicates using FLUOstar Omega microplate reader (BMG LABTECH). The data was analyzed using Omega MARS software and LDH release from cells was normalized to total LDH, obtained by lysing cells with 1% triton-X.

### Data analysis

Statistical significance was evaluated using the unpaired two-tailed Student’s *t*-test for comparing difference between two samples and one-way ANOVA for multiple comparisons. All data points or bars are presented as mean ± SEM. The significance level was set at *P* < 0.05.
